# Supplementary material for: Multi‐dataset identification of innovative feature genes and molecular mechanisms in keratoconus
Source: J Cell Mol Med. 2024 Sep 19;28(18):e70079. doi: 10.1111/jcmm.70079 (PMC11412914; doi:10.1111/jcmm.70079)
Supplement: Supplementary file 2 — Material S2. [file JCMM-28-e70079-s002.zip › 7.GSEA analysis/6.SNORA74B/KEGG_ASCORBATE_AND_ALDARATE_METABOLISM.html]

Details for gene set KEGG\_ASCORBATE\_AND\_ALDARATE\_METABOLISM[GSEA]

|  || Dataset | SNORA74B.group.cls#h\_versus\_l |
| Phenotype | group.cls#h\_versus\_l |
| Upregulated in class | h |
| GeneSet | KEGG\_ASCORBATE\_AND\_ALDARATE\_METABOLISM |
| Enrichment Score (ES) | 0.27847266 |
| Normalized Enrichment Score (NES) | 0.75579417 |
| Nominal p-value | 0.8299595 |
| FDR q-value | 1.0 |
| FWER p-Value | 1.0 |
Table: GSEA Results Summary

  

Fig 1: Enrichment plot: KEGG\_ASCORBATE\_AND\_ALDARATE\_METABOLISM      
 Profile of the Running ES Score & Positions of GeneSet Members on the Rank Ordered List

  

| SYMBOL | TITLE | RANK IN GENE LIST | RANK METRIC SCORE | RUNNING ES | CORE ENRICHMENT || 1 | UGT1A8 | na | 321 | 0.680 | 0.1889 | Yes |
| 2 | UGT1A6 | na | 2066 | 0.366 | 0.2452 | Yes |
| 3 | UGT2B4 | na | 3661 | 0.272 | 0.2785 | Yes |
| 4 | UGT2B11 | na | 6088 | 0.190 | 0.2639 | No |
| 5 | UGT2B28 | na | 7492 | 0.149 | 0.2668 | No |
| 6 | UGT2B10 | na | 9771 | 0.097 | 0.2294 | No |
| 7 | ALDH3A2 | na | 13104 | 0.046 | 0.1468 | No |
| 8 | UGT2A3 | na | 13387 | 0.043 | 0.1511 | No |
| 9 | ALDH7A1 | na | 19848 | -0.027 | -0.0272 | No |
| 10 | UGT2A1 | na | 21014 | -0.040 | -0.0490 | No |
| 11 | UGDH | na | 21842 | -0.050 | -0.0583 | No |
| 12 | UGT2B15 | na | 25363 | -0.105 | -0.1293 | No |
| 13 | ALDH9A1 | na | 25479 | -0.107 | -0.1014 | No |
| 14 | ALDH1B1 | na | 27039 | -0.140 | -0.1054 | No |
| 15 | MIOX | na | 28180 | -0.170 | -0.0889 | No |
| 16 | UGT2B7 | na | 31877 | -0.316 | -0.1032 | No |
| 17 | ALDH2 | na | 34359 | -0.636 | 0.0106 | No |
Table: GSEA details [plain text format]

  

Fig 2: KEGG\_ASCORBATE\_AND\_ALDARATE\_METABOLISM      
 Blue-Pink O' Gram in the Space of the Analyzed GeneSet

  

Fig 3: KEGG\_ASCORBATE\_AND\_ALDARATE\_METABOLISM: Random ES distribution      
 Gene set null distribution of ES for **KEGG\_ASCORBATE\_AND\_ALDARATE\_METABOLISM**
